# Digital food environments and youth diet: What’s next for school nutrition policies?

**DOI:** 10.1371/journal.pdig.0001540

**Published:** 2026-07-24

**Authors:** Mika Matsuzaki, Emma Sanchez-Vaznaugh, Joel Gittelsohn, Jessica Fanzo

**Affiliations:** 1 Department of International Health, Center for Human Nutrition, Johns Hopkins Bloomberg School of Public Health, Baltimore, Maryland, United States of America; 2 Department of Public Health and Health and Equity Institute, San Francisco State University, San Francisco, California, United States of America; 3 Center for Health Equity, University of California San Francisco, San Francisco, California, United States of America; 4 School of Advanced International Studies, Johns Hopkins University, Bologna, Italy; Yale University, UNITED STATES OF AMERICA

In recent years, *digital food environments* have proliferated globally and become a crucial aspect of food environments [1]. Digital food environments encompass the online spaces, platforms, and technologies through which food-related information, marketing and sales, and social and economic interactions occur [2]. These environments are increasingly influential in shaping dietary behaviors among youth, who are highly engaged with digital media and frequently exposed to persuasive food marketing content [3]. A recent report showed that 70% of Gen Z users identified social media as their primary tool for food discovery and restaurant information [4]. Online applications are also widely used across generations for food delivery, especially since the start of the COVID19 pandemic. In the United States (U.S.), spending on third-party delivery more than tripled for quick-service restaurants and quadrupled for full-service restaurants between 2019 and 2022 [5]. Algorithms and targeted advertising amplify exposure to energy-dense, nutrient-poor foods, while peer-generated content and influencer endorsements further normalize and reinforce unhealthy consumption patterns [6]. With the growing concerns around digital food environments, in 2021, the World Health Organization European Office for the Prevention and Control of Noncommunicable Diseases proposed a framework to tackle issues related to exposure to online marketing of unhealthy food and beverage products [2].

However, the digitization of the food environment has received disproportionately little attention in school nutrition policies and regulations, despite the increases in exposure to digital food environments during school hours. In our U.S. national survey in 2025, 1 in 4 teenagers reported having used mobile apps to order food during school hours [7]. Given the pervasiveness of youth interactions with digital food environments, this is an opportune time to assess how schools, students, and parents should approach various aspects of the digital food environments and inform the future national guidelines and policies. Many nations around the world have enacted laws to set the nutritional standards for foods and beverages served within schools, such as the federal Healthy, Hunger-Free Kids Act (HHFKA 2010) in the U.S. [8,9]. Studies have suggested the beneficial impact of these school nutrition policies—focusing largely on physical food environments within schools—on childhood obesity trends [10,11] as well as adverse effects of unhealthy physical food environments near schools on youth dietary behaviors and health outcomes [12].

Building upon these works, we must now ask: how should we extend these policies to include the digital food environments? What can be done to ensure healthy eating for schoolchildren in these rapidly shifting food environments? One approach is to limit or ban interactions with the digital food environments in school settings by restricting mobile phone use completely or to order food [13,14] or alternatively, limiting food orders from certain outlets that offer unhealthy foods and beverages. However, digital food culture is deeply embedded into the lives of the younger generations, and restrictions alone may not be as effective for ensuring healthy dietary intake among youth in the long run. A systematic review on randomized controlled trials with online platform interventions found that increased visibility of nutrition information and healthy foods showed promising results on the reduction of total calories, fat, saturated fat, and sodium content of online food purchases [15]. These results present a case for considering alternative approaches to managing digital food environments for schools, school districts, and governmental offices. For instance, it may be useful to explore partnerships with the delivery app companies and food outlets that use those apps to promote healthier choices for youth when they order items. Another approach is curricular innovations. Schools could be an excellent venue to equip the majority of the children and adolescents with the knowledge and skills needed to make intentional smart choices with tools and options offered through physical and digital food environments. This type of skills and knowledge can be applied beyond school settings and after graduation. Financial incentives for making healthier choices, such as point cards or discounts on healthier items, may also be appealing to adolescents with limited budgets to avoid unhealthy food/drinks.

Food environments surrounding schoolchildren will continue to change. Digital food environments present challenges and opportunities in promoting healthy diets through policies and regulations. The sole focus on physical food environments in school nutrition policies may soon become obsolete and less effective in changing dietary behaviors. The expansion of school nutrition policies should involve conversations with all levels of “digital actors”, from state and local governments, the food industry, the media, to individual influencers and users [1]. Youth perspectives are important as they are often more experienced in and kept abreast of the latest technologies and may offer useful insights into the food systems, utility, and social impact that policymakers may not be aware of. To support development of policies to help youth adapt to the ongoing—and future—shift in food environments, we should strengthen the evidence base and build a new conceptual and behavior change framework for examining the combined impact of physical and digital food environments on food and nutrition choices and health outcomes among youth. Multi-component, multi-level intervention trials in diverse settings and subpopulations using both online platforms and physical venues are essential to improving dietary behaviors, knowledge, and perceptions among schoolchildren through the next set of school nutrition policies.
